# A Time-Varying Seasonal Autoregressive Model-Based Prediction of Respiratory Motion for Tumor following Radiotherapy

**DOI:** 10.1155/2013/390325

**Published:** 2013-06-10

**Authors:** Kei Ichiji, Noriyasu Homma, Masao Sakai, Yuichiro Narita, Yoshihiro Takai, Xiaoyong Zhang, Makoto Abe, Norihiro Sugita, Makoto Yoshizawa

**Affiliations:** ^1^Department of Electrical and Communication Engineering, Graduate School of Engineering, Tohoku University, 6-3 Aoba, Aramaki, Aoba-ku, Sendai, Miyagi 980-8578, Japan; ^2^Division on Radiological Imaging and Informatics, Tohoku University Graduate School of Medicine, Seiryo-machi, Aoba-ku, Sendai, Miyagi 980-8578, Japan; ^3^Division for Media Education, Center for Information Technology in Education, Tohoku University, 41 Kawauchi, Aoba-ku, Sendai, Miyagi 980-8576, Japan; ^4^Division for Radiology and Radiation Oncology, Hirosaki Univeristy School of Medicine, Hirosaki, 5 Zaifu-cho, Hirosaki, Aomori 036-8562, Japan; ^5^Department of Electrical Engineering, Graduate School of Engineering, Tohoku University, 6-3 Aoba, Aramaki, Aoba-ku, Sendai, Miyagi 980-8578, Japan; ^6^Department of Management of Science and Technology, Graduate School of Engineering, Tohoku University, 6-3 Aoba, Aramaki, Aoba-ku, Sendai, Miyagi 980-8578, Japan; ^7^Research Division on Advanced Information Technology, Cyberscience Center, Tohoku University, 6-3 Aoba, Aramaki, Aoba-ku, Sendai, Miyagi 980-8578, Japan

## Abstract

To achieve a better therapeutic effect and suppress side effects for lung cancer treatments, latency involved in current radiotherapy devices is aimed to be compensated for improving accuracy of continuous (not gating) irradiation to a respiratory moving tumor. A novel prediction method of lung tumor motion is developed for compensating the latency. An essential core of the method is to extract information valuable for the prediction, that is, the periodic nature inherent in respiratory motion. A seasonal autoregressive model useful to represent periodic motion has been extended to take into account the fluctuation of periodic nature in respiratory motion. The extended model estimates the fluctuation by using a correlation-based analysis for adaptation. The prediction performance of the proposed method was evaluated by using data sets of actual tumor motion and compared with those of the state-of-the-art methods. The proposed method demonstrated a high performance within submillimeter accuracy. That is, the average error of 1.0 s ahead predictions was 0.931 ± 0.055 mm. The accuracy achieved by the proposed method was the best among those by the others. The results suggest that the method can compensate the latency with sufficient accuracy for clinical use and contribute to improve the irradiation accuracy to the moving tumor.

## 1. Introduction

In radiation therapy, some internal organ motions can make critical misalignment between irradiated field and the target volume during a treatment fraction. For example, a lung tumor can move over a centimeter per second mainly due to patients' respiration [1].

A real-time image-guided technique can be used for managing such tumor motion [2]. Indeed, kV X-ray fluoroscopes [3–5] and electronic portal imaging devices (EPIDs) [6] have been developed for monitoring the intrafractional tumor motion in real time. The measured tumor position is then potentially used for targeting the irradiation field by using a dynamic multileaf collimator (dMLC) [7]. In the real-time targeting system, the exposure to normal tissues can be reduced, and thus the dose rate escalation can be allowed for better treatment results. The system takes, however, up to several hundred milliseconds to control the irradiation field by using dMLC after the moment of measuring the tumor position [8]. Obviously such time delay causes a misalignment between the controlled isocenter and the center of the moving target volume.

A general and useful way to compensate the time delay is to predict respiratory-induced tumor motion [9], and many prediction methods of respiratory motion have been proposed [10]. In fact, there are many approaches for predicting respiratory motion, such as linear and nonlinear regression models [11, 12], neural networks [13–15], autoregressive moving-average models [16, 17], probabilistic approaches [18], and singular spectrum analyses [19]. However, to the best of our knowledge, no approach that can satisfy clinical requirements in the sense of prediction accuracy has yet been developed due to the complexity of the respiratory motion.

Even the respiratory motion is very complex it is not surprising that a fundamental pattern involved in the tumor motion is periodical behavior because respiration consists of repetition of inhaling and exhaling alternately. The periodicity tells us much information about what the future will be because the current state will arise again after a certain period of time. This periodic nature can be useful for the motion prediction, but the time varying and fluctuated periodicity, or quasi-periodicity, involved in the respiratory motion remain as an impediment to periodicity-based prediction.

To model the time-varying periodical nature of respiration, periodic autoregressive moving-average (PARMA) [16] and modified seasonal autoregressive integrated moving-average (SARIMA) [17] approaches have been proposed. The PARMA model-based method decomposes the time series into two components: a fully periodic component consisting of an average wave form and the other component. However, it is unclear how to extract the periodical component. Also, fast response of the model adaptation to the fluctuation of the periodicity might be difficult due to the hysteresis in the calculation of the average wave form for the periodic component extraction. On the other hand, the SARIMA model-based method converts the time-varying periodic nature to a constant periodic one by adjusting the time variation. However, the model cannot express the target time series with desirable accuracy unless the conversion is perfect, which is very difficult in general. Consequently, these time-varying periodical models need to be improved to achieve better performance with sufficient prediction accuracy.

In this paper, to improve the prediction accuracy, we develop a new prediction method by taking into account the periodical respiratory nature with complex fluctuation observed in the lung tumor motion. The goal is to predict the tumor motion at several hundred milliseconds ahead with a high accuracy less than 1 mm, so that a minimal requirement for irradiation with submillimeter accuracy [20] would be satisfied. The proposed model is based on a seasonal autoregressive model, but newly designed to adapt to the fluctuated periodicity by using a correlation analysis-based methodology. The prediction performance of the proposed method is evaluated by using clinical data sets.

The outline of this paper is as follows. In the next section, the target motion of a lung tumor is analyzed briefly. Based on the analysis, a new time-varying seasonal autoregressive model is proposed in Section 2.2. In Section 3, experimental details including clinical data sets are tested, and evaluation index for prediction performance is described. Prediction results by using the clinical data sets are discussed in Section 4. The last section provides concluding remarks.

## 2. Methods and Materials

### 2.1. Time Series of Lung Tumor Motion

**Figure 1:**
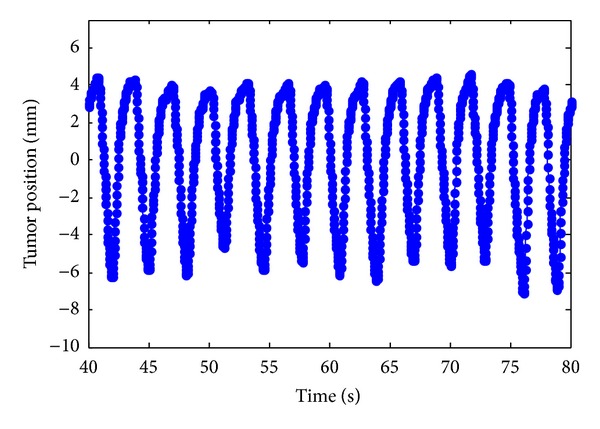
A part of time series of a lung tumor motion. The position of the tumor periodically changes with time by patient's respiration.

 Let us consider a time series of a lung tumor motion as shown in Figure 1, denoted by {*y*(*t*)}, *t* = 1,2,…. Here *y*(*t*) is a coordinate of the tumor position at discrete time index *t*.

It is apparent that the lung tumor position changes almost periodically with some amplitude variation. It can be found that the tumor returns to the same position or its neighbor every 3 s (90 samples in this case) in average. It was however confirmed that time intervals between positive/negative peaks and their preceding or following positive/negative peaks are not constant but time varying, even if the amplitudes of the positive/negative peaks are almost similar to each other.

It may be worth to mention that the periodical nature involves complex fluctuation even the fact that the lung tumor moves almost periodically is very useful information for predicting the motion.

### 2.2. Prediction Methods

#### 2.2.1. Seasonal Autoregressive Model-Based Prediction

 Seasonal autoregressive (SAR) model is an autoregressive (AR) model to express time series with periodical or seasonal variation [21]. The SAR model of time series {*y*(*t*)}, *t* = 1,2,… is given as follows:

(1) $$\begin{array}{c} y\left(t\right)=\epsilon \left(t\right)+\sum_{\rho =1}^{P}\Phi_{\rho }\cdot y\left(t-\rho \cdot s\right), \end{array}$$

where Φ_*ρ*_, *ρ* = 1,2,…, *P* are SAR coefficients, *P* is the order of SAR model, and *ϵ*(*t*) is a white Gaussian noise with mean *μ* = 0 and variance *σ* ^2^ at time *t*, respectively. Note that *s* is a period such that a time series of {*y*(*t*), *y*(*t* − *s*),…} is represented by the *P*th autoregressive model [21]. The period can be estimated based on an analysis of the past samples available at the current time *t*, {*y*(*t* − 1), *y*(*t* − 2),…}. One may use the autocorrelation analysis and Fourier analysis for such estimation. Then, for *h* ∈ {1,2,…}, the *h*-sample forward prediction can be obtained by substituting *t* + *h* for *t* in (1) as follows:

(2) $$\begin{array}{c} \hat{y}\left(t+h\;|\;t\right)=\sum_{\rho =1}^{P}{\hat{\Phi }}_{\rho }\cdot y\left(t+h-\rho \cdot s\right). \end{array}$$

Here, $\hat{y}(t+h|t)$ denotes an estimate of *y*(*t* + *h*) at time *t*, and ${\hat{\Phi }}_{\rho }$, *ρ* = 1,2,…, *P* are estimates of Φ_*ρ*_. Note that *h* in the right side of (2) must not be greater than *ρ* · *s*, that is, *h* ≤ *ρ* · *s*, to predict the future value using only the past values.

As is clear from (1) and (2), the SAR model predicts the target value *y*(*t* + *h*) as the weighted sum of *P* past values at *ρ*th (*ρ* = 1,2,…, *P*) period samples back.

An apparent problem here is that the SAR model inherently assumes the constant period *s* which is not time varying. In other words, the constant period *s* of the SAR model cannot accurately refer to the past values correlated to the prediction target if the target time series involves quasi-periodic nature. This mismatch has a bad effect on the prediction accuracy. In this sense, the SAR model is not suitable for predicting time-varying periodic respiratory motion.

#### 2.2.2. Time-Varying SAR Model-Based Prediction

 To overcome the limitation of the conventional SAR model-based method, a time-varying interval is introduced instead of the constant period *s* for referring to the past values correlated to the prediction target. In the following, let us keep considering the linear regression approach of SAR model. However, other approaches such as nonlinear adaptive filtering can be incorporated into the proposed concept of time-varying interval model.

*(a) Basic Concept of Time-Varying SAR Model*. A time-varying SAR (TVSAR) model is defined as follows:

(3) $$\begin{array}{c} y\left(t\right)=\epsilon \left(t\right)+\sum_{\rho =1}^{P}\Phi_{\rho }\cdot y\left(t-r_{\rho }\left(t\right)\right), \end{array}$$

where *r*_*ρ*_(*t*) > 0, *ρ* = 1,2,…, *P* are the *ρ*th reference intervals by which the target value *y*(*t*) is matched with the past values at the reference intervals *r*_*ρ*_(*t*)-sample back, {*y*(*t* − *r*_*ρ*_(*t*))}.

For a time series with a constant period *s*, the reference intervals are given as *r*_*ρ*_(*t*) = *ρ* · *s*. Thus, the conventional SAR model in (1) can be regarded as a special case of the model in (3). In other words, the time-varying SAR model is an extension of the conventional SAR model for adapting to a time-varying periodical nature.

*(b) Reference Interval Estimation*. How to estimate the reference intervals is a fundamental problem to be solved for building the time-varying SAR model. In this study, a correlation analysis-based approach was adopted for estimating the reference intervals. That is, the intervals are estimated based on the best match between the target and past subsets of the time series in the sense of the correlation.

The correlation function CF between the target subset at time *t*, [*y*(*t* − *w* + 1), *y*(*t* − *w*),…, *y*(*t*)], and *k*-sample lagged subset, [*y*(*t* − *k* − *w* + 1), *y*(*t* − *k* − *w*),…, *y*(*t* − *k*)], is given as follows:

(4) $$\begin{array}{c} \text{CF}\left(t,k\right)=\frac{1}{w}\sum_{j=0}^{w-1}\frac{y\left(t-j\right)-\mu_{t}}{\sigma_{t}}\cdot \frac{y\left(t-k-j\right)-\mu_{t-k}}{\sigma_{t-k}},\; \end{array}$$

where *μ*_*t*_ and *σ*_*t*_ are the sample mean and standard deviation of the subset at time *t*, respectively. Then, the estimates of the *ρ*th reference interval ${\tilde{r}}_{\rho }(t)$ are obtained as the interval from lag *k* = 0 to the *ρ*th positive peak of the correlation function

(5) $$\begin{array}{c} {\tilde{r}}_{\rho }\left(t\right)=\arg\underset{k\in K_{\rho }}{\max}\;\text{CF}\left(t,k\right), \end{array}$$

where *K*_*ρ*_, *ρ* ∈ {1,2,…, *P*} determines a search range for the *ρ*th intervals and is set as $K_{\rho }=\left\{k|{\tilde{r}}_{\rho }(t-1)-{\tilde{r}}_{1}(t-1)/2\leq k\leq {\tilde{r}}_{\rho }(t-1)+{\tilde{r}}_{1}(t-1)/2\right\}$.

The subset-length *w* affects the sensitivity of the estimation. The shorter length of subset can follow the quicker change of the reference interval, while it may lack the more information for measuring the similarity and be the more susceptible to noise. The longer subset can cover the larger number of the sample values to evaluate the similarity, while it can follow the slower internal change. In this sense, *w* should be determined by balancing the amount of information for similarity estimation against the response speed. It is rational to assume that at least a cycle-length subset may be needed to estimate the reference interval that implies a cycle length. Since the estimate of the first reference interval ${\tilde{r}}_{1}(t)$ is expected to cover an approximate full length of the current respiratory cycle, *w* is updated as ${\tilde{r}}_{1}(t)$ each time in this paper. That is, $w={\tilde{r}}_{1}(t)$.

The effect of the past information can be reduced by using information from the shorter-length subset. Especially, the point-to-point analysis uses the shortest subset of *w* = 1 implying a value *y*(*y*). To compensate the lack of information due to the short length, not only the values of a subset, but also the derivatives can be used. Then, the correlation analysis-based estimates of reference intervals ${\tilde{r}}_{\rho }(t)$ will be adjusted by the point-to-point analysis of the value and the first derivatives in this paper.

The adjustment procedure is as follows.

(1) Estimate the reference intervals, ${\tilde{r}}_{\rho }(t)$ by using the correlation analysis-based approach.
(2) Evaluate the difference between the current and past samples around $y(t-{\tilde{r}}_{\rho }(t))$. The evaluation function is given as
  (6) Fρ(l)=α(y(t+l−r~ρ(t))−y(t))2+β(sgn⁡(y˙(t+l−r~ρ(t))−y˙(t)))2,
 where *l* is a lag from $t-{\tilde{r}}_{\rho }(t)$, *α* and *β* are coefficients for the nondimensionalization, sgn⁡(·) is a signum function, and $\dot{y}(t)$ denotes the first derivative of *y*(*t*) and is approximated by $\dot{y}(t)=\left(1/3\right)\sum_{j=1}^{3}\left\{y(t-j)-y(t-j-1)\right\}$.
(3) Find the local minimum of the evaluation function *F*_*ρ*_(*l*) and then obtain an amount of adjustment Δ*r*_*ρ*_ as
  (7) $$\begin{array}{c} \Delta r_{\rho }=\arg\underset{-L\leq l\leq +L}{\min}F_{\rho }\left(l\right), \end{array}$$
 where *L* determines a range of adjustment.
(4) Adjust the correlation analysis-based estimates by using Δ*r*_*ρ*_ as
  (8) $$\begin{array}{c} {\overset{ˇ}{r}}_{\rho }\left(t\right)={\tilde{r}}_{\rho }\left(t\right)+\Delta r_{\rho }, \end{array}$$
 where ${\overset{ˇ}{r}}_{\rho }(t)$, *ρ* = 1,2,…, *P* are new estimates.

Thus, the adjusted estimates ${\overset{ˇ}{r}}_{\rho }(t)$, *ρ* = 1,2,…, *P* can refer to the past values which are feasible to predict the current one *y*(*t*) in terms of amplitude and velocity.

*(c) Prediction Equations Based on TVSAR Model*. For the *h*-sample ahead prediction, the proposed time-varying SAR model can be represented by substituting *t* + *h* for *t* in (3) as

(9) $$\begin{array}{c} \hat{y}\left(t+h\;|\;t\right)=\sum_{\rho =1}^{P}{\hat{\Phi }}_{\rho }\cdot y\left(t+h-r_{\rho }\left(t+h\right)\right). \end{array}$$

Here, the reference intervals at the *h*-sample future, *r*_*ρ*_(*t* + *h*), are unknown values. Therefore, the prediction equation above is rewritten in practice as follows:

(10) $$\begin{array}{c} \hat{y}\left(t+h\;|\;t\right)=\sum_{\rho =1}^{P}{\hat{\Phi }}_{\rho }\cdot y\left(t+h-{\hat{r}}_{\rho }\left(t+h\;|\;t\right)\right), \end{array}$$

where ${\hat{r}}_{\rho }(t+h|t)$, *ρ* = 1,2,…, *P* denote the reference interval estimates at time *t* + *h*, predicted at the current time *t*. Note that reference intervals must be greater than prediction horizon *h*, that is, $h\leq {\hat{r}}_{\rho }(t+h|t)$, to compose the prediction using the past values.

Then, we have two types of reference intervals, correlation analysis-based and its adjusted estimates, as mentioned earlier; thus the following two types of TVSAR model-based prediction equations are introduced. 

*TVSAR(a)—Prediction with Correlation Analysis-Based Reference Interval*. If we adopt the zero order hold of the correlation-based reference intervals ${\tilde{r}}_{\rho }(t)$ in (5) as the prediction

(11) $$\begin{array}{c} {\hat{r}}_{\rho }\left(t+h\;|\;t\right)={\tilde{r}}_{\rho }\left(t\right) \end{array}$$

then the prediction equation based on TVSAR is given as

(12) $$\begin{array}{c} \hat{y}\left(t+h\;|\;t\right)=\sum_{\rho =1}^{P}{\hat{\Phi }}_{\rho }\cdot y\left(t+h-{\tilde{r}}_{\rho }\left(t\right)\right). \end{array}$$

*TVSAR(b)—Prediction with Adjusted Reference Interval*. Similarly, if we adopt the zero order hold of the adjusted reference interval estimate ${\overset{ˇ}{r}}_{\rho }(t)$ in (8) as the prediction

(13) $$\begin{array}{c} {\hat{r}}_{\rho }\left(t+h\;|\;t\right)={\overset{ˇ}{r}}_{\rho }\left(t\right) \end{array}$$

then TVSAR model-based prediction is given as

(14) $$\begin{array}{c} \hat{y}\left(t+h\;|\;t\right)=\sum_{\rho =1}^{P}{\hat{\Phi }}_{\rho }\cdot y\left(t+h-{\overset{ˇ}{r}}_{\rho }\left(t\right)\right). \end{array}$$

## 3. Experimental Setup

 We have evaluated the prediction performance of the proposed method by using some clinical data sets.

### 3.1. Prediction Methods

 For comparison, the following methods including the-state-of-the-art ones were tested on the data sets:

- zero order hold (ZOH);
- singular spectrum analysis (SSA) based method [19];
- kernel density estimation (KDE) based method [18];
- adaptive SAR model-based method [17]:
  - adaptive SAR is given as (2) by substituting $\hat{s}(t+h|t)={\tilde{r}}_{1}(t)$ for *s*;
- time-varying SAR (TVSAR) model-based method (proposed):
  - reference interval estimates based on correlation analysis;
  - adjusted reference interval estimates.

Table 1 summarizes the experimental settings used for the performance evaluation. These are based on the original settings and partially modified to obtain better performance for the data sets.

### 3.2. Data Sets of Lung Tumor Motion

#### 3.2.1. Original Data Sets

**Figure 2:**
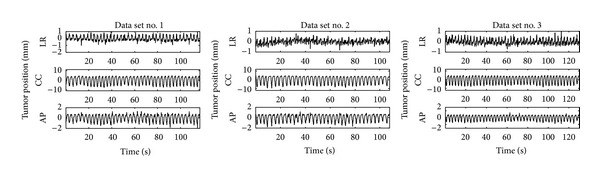
Three data sets of lung tumor motion. The vertical-axis labels LR, CC, and AP stand for lateral-axis, cephalocaudal-axis, and anteroposterior-axis, respectively.

 Three data sets of lung tumor motion acquired at Hokkaido University Hospital were used for the evaluation. The three-dimensional lung tumor positions were measured as the trajectory of gold fiducial markers implanted near the tumor, by using X-ray fluoroscopic system with sampling rate of 30 Hz. To eliminate the outliers and high-frequency noise in each time series, low-pass and statistical filters were used preliminarily for all the data sets. The three data sets used in this paper are shown in Figure 2.  Table 2 summarizes the characteristics of each data set.

#### 3.2.2. Data Sets with Lower Sampling Rate

There are several systems for measuring or estimating the tumor motion, such as CCD camera systems with chest markers and fluoroscopic imaging systems with implanted golden markers. These imaging systems have a variety of sampling rates, and actual sampling rate in clinical use may be less than or equal to the maximum sampling rate of the device in order to suppress the additional radiation exposure. To evaluate the effect of the sampling rate on the prediction performance, data sets with the lower sampling rates *F*_*s*_ = 5, 10, 15, 20, and 25 Hz were generated from the original data sets and used for this experiment.

The problem here is that the lower rate provides only the lower pieces of information about the tumor motion, and thus it may badly affect the prediction accuracy. To avoid the bad effects of low sampling rates, online interpolation using cubic-spline was adopted as a preprocessing for the prediction methods. Using the interpolation, any low sampling rate less than 30 Hz was upsampled to 30 Hz in this evaluation.

### 3.3. Evaluation Index for Prediction Performance

We evaluate the prediction accuracy by using mean absolute error (MAE) given as a function of the prediction horizon *h*,

(15) $$\begin{array}{c} \text{MAE}\left(h\right)=\frac{1}{t_{e}-t_{s}}\sum_{t=t_{s}}^{t_{e}}\left|e_{\text{euc}}\left(t\;|\;t-h\right)\right|. \end{array}$$

Here *t*_*s*_ and *t*_*e*_ are, respectively, the lower and upper bounds for defining the evaluation interval, and *e*_euc_(*t* | *t* − *h*) is the Euclidean distance between the predicted and actual positions given by

(16) $$\begin{array}{c} e_{\text{euc}}\left(t\;|\;t-h\right)=\sqrt{\sum_{i}{\left({\hat{y}}_{i}(t\;|\;t-h)-y_{i}(t)\right)}^{2}}, \end{array}$$

where *i* = {LR, CC, and  AP} are indices for three-dimensional space and correspond to lateral-, cephalocaudal-, and anteroposterior-axes, respectively.

The lower bound was fixed as *t*_*s*_ = 551 (approximately 18.4 s), and the upper bounds were determined as *t*_*e*_ = *T* − *h*_max⁡_, where *T* denotes the length of the time series, and *h*_max⁡_ = 30 is the maximum prediction horizon. Then, for the data sets of no. 1, no. 2, and no. 3, *t*_*e*_ = 3470, 3198, and 3870 (approximately 115.7, 106.6, and 129.0 s), respectively.

Generally, MAE increases when increasing prediction horizon *h* becomes large, but it is required to be less than 1 mm at prediction horizon of several hundred milliseconds at least for dMLC tracking with submillimeter accuracy [20].

## 4. Results and Discussions

### 4.1. Prediction with the Full Sampling Rate

**Figure 3:**
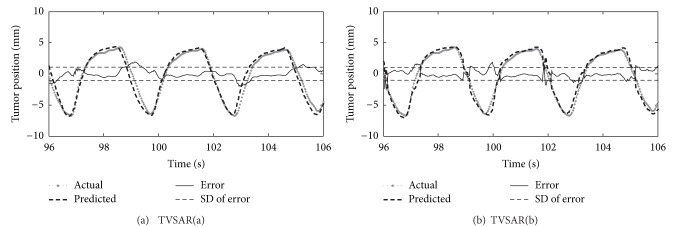
An example of 0.5 s forward predictions on the data set no. 1 at *F*_*s*_ = 30 Hz. (a) TVSAR with amplitude-based reference interval estimation. (b) TVSAR with correlation-based reference interval estimation.

Figure 3 shows selected examples of one-dimensional time series from the cephalocaudal-axis (*i* = CC) of data sets no. 1 predicted by the proposed method for *h* = 15 samples ahead (i.e., 0.5 s future). Gray dots, dashed, solid, and dotted lines depict the actual position, the position predicted, the error $e(t|t-h)=\hat{y}(t|t-h)-y(t)$, and the standard deviation of the error *e*(*t* | *t* − *h*), *t* = *t*_*s*_,…, *t*_*e*_, respectively.

According to the figures, it can be seen that tumor positions predicted by TVSAR(a) and (b) are close to the actual one in appearance. TVSAR(a) prediction is smoother than TVSAR(b) prediction, and this fact suggests that TVSAR(a) is better than TVSAR(b) from a viewpoint of the smoothness. On the other hand, the averages and standard deviations of prediction errors by TVSAR(a) and (b) were −0.0667 ± 1.0677 mm and −0.0043 ± 1.0291 mm, respectively. Both are almost within 1 mm accuracy, but TVSAR(b) is slightly better than (a).

The error of TVSAR(b), which is peaky, but smaller than TVSAR(a) on average, might be caused as the result of the adjustment of the reference interval. Indeed, once the difference between the current value *y*(*t*) and its predicted value $y(t-{\tilde{r}}_{\rho }(t))$ of TVSAR(a) becomes large, the reference interval estimate of TVSAR(b) is adjusted to reduce the difference by using past values. Consequently, large errors of TVSAR(a) are basically suppressed by TVSAR(b).

**Figure 4:**
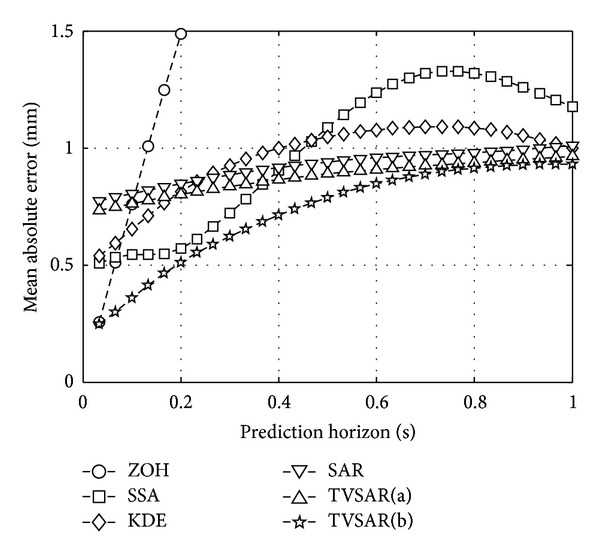
Averaged MAEs of tested prediction methods at sampling frequency *F*_*s*_ = 30 Hz.

For three-dimensional performance evaluation with other prediction methods, Figure 4 shows MAE averaged over the three clinical data sets, as a function of prediction horizon *h*/*F*_*s*_ (s). Also, Table 3 summarizes the averages and the standard deviations of MAEs achieved by the prediction methods at selected prediction horizons of *h* = 5, 10, 15, 20, 25, and 30. The best MAEs for each prediction horizon are indicated by boldface.

As shown in Figure 4 and Table 3, the MAEs of the two types of the proposed methods are less than 1 mm for *h*/*F*_*s*_ ≤ 1 s. The least MAE for 1 ≤ *h* ≤ 30 was achieved by TVSAR(b) in this experiment. On the other hand, other prediction methods presented that those MAEs are larger than 1 mm, at each different prediction horizon. The MAE of ZOH is very small at *h* = 1 but has drastically increased over 1 mm for *h* > 3. The three methods of KDE, SSA, and the adaptive SAR showed less MAE than ZOH except for the very short-term prediction. However, those MAE curves were over 1 mm for *h*/*F*_*s*_ > 0.4 s. The MAE curve of the SAR is very flat and seems similar to TVSAR(a) but is slightly larger than that of TVSAR(a). This may be because SAR and TVSAR(a) share those first past values used for their predictions, that is, $\hat{y}\left(t+h-\hat{s}\left(t+h|t\right)\right)=y\left(t+h-{\hat{r}}_{1}\left(t+h|t\right)\right)$.

As shown in the result of MAE, the two proposed methods can predict the tumor motion with the order of submillimeter accuracy on average. In addition to this, it was shown that the prediction accuracy of TVSAR(b) is the best among the compared methods including TVSAR(a). Especially, only TVSAR(b) is superior to ZOH for very short-term prediction of *h*/*F*_*s*_ < 0.1 s. The reference interval adjustment method used for TVSAR(b) can decrease the prediction error at short- and mid-term predictions, and there is no apparent negative effect for long-term prediction.

In summary, the proposed TVSAR(a) and (b) can predict the lung tumor motion at up to 1 s future with the order of submillimeter on average. The amplitude-based reference interval adjustment used for TVSAR(b) is useful to decrease prediction error at short- and mid-term prediction. These indicate that the concept of TVSAR plays an important role to adapt to the fluctuated periodicity and to efficiently use the past values similar to the current value as accurate as possible by capturing time-varying periodical nature.

As reported in previous studies [1, 18], there may be clinical data of the tumor motion with larger trend and amplitude variation compared to those of data sets used in this paper. For such complex motions, the proposed adjustment might provide reference interval estimates with insufficient accuracy. This is because the proposed method does not carefully take into account the trend and amplitude variation. However, it is expected that the trend can be included in the model as additional components such as integral operators, and the amplitude variation can be followed by designing the SAR coefficients. These refinements of the proposed method can contribute to further improvement of the prediction performance.

It has also been reported that audiovisual biofeedback can make breathing pattern stable and improve accuracy of KDE-based prediction [22, 23]. As shown in the results, TVSAR is superior to KDE for respiratory motion with relatively regular pattern. Consequently, a combination of the biofeedback technique and TVSAR can improve the prediction performance for various patients.

### 4.2. Prediction with Low Sampling Rates

**Figure 5:**
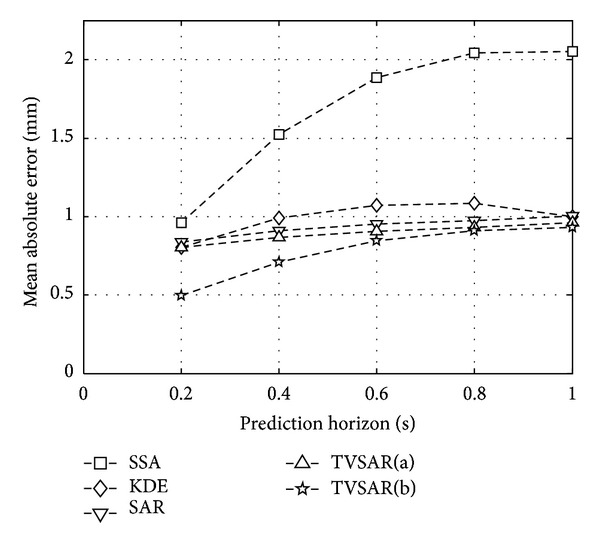
Mean absolute errors of tested prediction methods at sampling frequency *F*_*s*_ = 5 Hz.

 To evaluate the effect of lower sampling rates on the prediction performance, MAEs for prediction of *h*/*F*_*s*_ = 0.2, 0.4, 0.6, 0.8, and 1.0 s future of the tumor positions sampled at sampling rate of *F*_*s*_ = 5 Hz are shown in Figure 5. The performances of ZOH here were almost the same as the full sampling rate case shown in Figure 4 and thus omitted in this evaluation.

As shown in the both figures, the most prediction performances for lower rates were rather equivalent to those for the full sampling rate. For example, at 1 s ahead prediction for *F*_*s*_ = 5 Hz, MAEs of the proposed methods were less than 1 mm.

**Figure 6:**
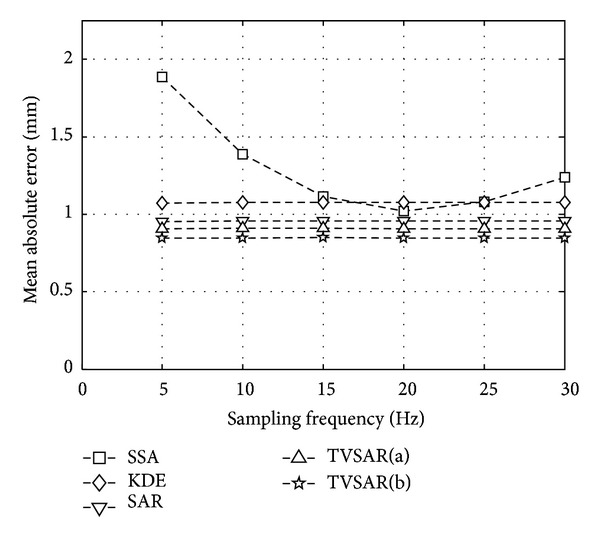
Mean absolute errors of tested prediction methods as a function of sampling frequency *F*_*s*_ (Hz) at *h*/*F*_*s*_ = 0.6 s.

Figure 6 summarizes MAEs for prediction of *h*/*F*_*s*_ = 0.6 s future positions sampled at several sampling rates *F*_*s*_ = 5, 10, 15, 20, 25, and 30 Hz. According to the results for the sampling frequencies *F*_*s*_ = 5, 10, 15, 20, 25, and 30 Hz, the two proposed methods achieved higher performance than others. KDE and SAR achieved the same accuracies for lower sampling rates with the online interpolation, but these are lower than the proposed methods. The accuracy of SSA on lower rates was worse than that for the full sampling rate case. This is because there were differences between the eigenvalues used for SSA obtained from the interpolated time series and those obtained from the time series observed by the full rate. This presents that the interpolated time series are missing important information required for the SSA-based prediction.

Generally, prediction performance decreases as sampling rate decreases because time series with lower sampling rates have much smaller pieces of information to be used for prediction. However, most of prediction performances for various sampling rates have not been changed very much by the online interpolation used in this study. This suggests that the online interpolation works well to suppress the bad effect of lower sampling rates on prediction accuracy. At the same time, the change of SSA's performance suggests that unsuitable combination of lower sampling rates and the prediction method may cause larger irradiation error. Thus, the interpolation, with a feasible prediction method, can be useful to decrease the sampling rate of X-ray fluoroscopic imaging system for suppressing the additional exposure.

In this study, the data sets were acquired by measuring the internal tumor position directly. On the other hand, an external respiratory signal such as the surface motion of the breast can be used as a surrogate signal of internal tumor motion. Indeed, such surrogate signal can be measured in an easier way than the direct measurement of the internal position. It can also avoid the side effect and thus has widely been used in actual treatment. However, there can be a large difference between the actual tumor position and the external surrogates for patients with significant phase shift as reported in the previous study [24].

## 5. Conclusions

 In this paper, a TVSAR model-based method for respiratory motion prediction was proposed for compensating time delay in radiotherapy devices. To adapt to the fluctuation, a time-varying interval was introduced for composing the prediction from the observed past motion. For adapting the interval to the time series, the correlation analysis-based method and the adjustment were also proposed. The proposed method was tested on clinical tumor motion data sets and compared to several other methods including the state-of-the-art ones. It has been demonstrated that the proposed method performed prediction within 1 mm accuracy at 1 s ahead on average. The result is superior to those of compared prediction methods. Especially, the proposed method with the adjustment of the interval achieved the least average error for a wide prediction horizon from 0.033 s to 1 s. This suggests that the internal margin on tumor following irradiation can be set within submillimeter by using the proposed method. Consequently, we may conclude that the proposed method can contribute to improve the irradiation accuracy on real-time tumor following radiation therapy.

## Figures and Tables

**Table 1:** Tested prediction methods and settings.

Method	Setting	
ZOH	None	—

	Samples back length	300 samples
SSA [19]	Dimension number of covariance matrix	250 samples
	Number of largest eigenvalues	18

	Sampling interval	15 samples
KDE [18]	Dimension number of covariate	3
	Length of moving window	300 samples
	Representing value of distribution	Mean

Adaptive SAR [17]	Order of seasonal AR	*P* = 2
	Coefficients of SAR	${\hat{\Phi }}_{\rho }=1/2$, *ρ* = 1,2,…, *P*

	Order of TVSAR	*P* = 2
TVSAR	Coefficients of TVSAR	${\hat{\Phi }}_{\rho }=1/2$, *ρ* = 1,2,…, *P*
(proposed method)	Reference interval estimation	(a) Correlation analysis-based approach
		(b) Adjustment with adjustment range *L* = 5

**Table 2:** Characteristics of data sets tested. The sampling frequency is 30 Hz for all the data sets.

	Axis	Case number		
		No. 1	No. 2	No. 3
Max. amplitude of respiration cycle max⁡|*y*(*t*)| (mm)	LR	1.1409 mm	0.8268 mm	1.1748 mm
	CC	7.6957 mm	9.4093 mm	8.3144 mm
	AP	1.9169 mm	1.7538 mm	1.7833 mm

Standard deviation of time series *σ*_*y*_ (mm)	LR	0.3351 mm	0.2064 mm	0.2475 mm
	CC	3.6548 mm	3.9511 mm	3.8863 mm
	AP	0.622 mm	0.6179 mm	0.6743 mm

Average period of breathing cycle $\overset{-}{s}/F_{s}$ (s)		3.0341 s	2.9681 s	3.0341 s

Length of time series (s)		116.7 s	107.6 s	130.0 s

**Table 3:** Average and standard deviation of mean absolute error for each tested prediction method at sampling frequency *F*_*s*_ = 30 Hz.

Prediction horizon		Average and standard deviation of mean absolute error *μ*_MAE_ ± *σ*_MAE_ (mm)					
*h*	*h*/*F*_*s*_ (s)	ZOH	SSA	KDE	SAR	TVSAR(a)	TVSAR(b)
5	0.167	1.248 ± 0.008	0.548 ± 0.051	0.764 ± 0.021	0.833 ± 0.034	0.790 ± 0.011	** 0.466** ± **0.017 **
10	0.333	2.394 ± 0.026	0.781 ± 0.072	0.953 ± 0.029	0.896 ± 0.021	0.846 ± 0.019	** 0.653** ± **0.016 **
15	0.500	3.446 ± 0.047	1.086 ± 0.094	1.047 ± 0.054	0.940 ± 0.011	0.889 ± 0.027	** 0.787** ± **0.037 **
20	0.667	4.387 ± 0.068	1.299 ± 0.099	1.087 ± 0.059	0.966 ± 0.010	0.918 ± 0.032	** 0.876** ± **0.047 **
25	0.833	5.188 ± 0.091	1.305 ± 0.066	1.078 ± 0.048	0.983 ± 0.018	0.941 ± 0.037	** 0.920** ± **0.052 **
30	1.000	5.834 ± 0.111	1.177 ± 0.039	0.999 ± 0.056	1.011 ± 0.021	0.965 ± 0.039	** 0.931** ± **0.055 **
